# Construction of Pt-Cu-Vinylamine Complex on Hazelnut Shell Biochar as a Catalyst Used for Hydrosilylation of Alkenes by Tertiary Silanes

**DOI:** 10.3390/molecules30183704

**Published:** 2025-09-11

**Authors:** Jing Zhou, Qiqi Zhang, Mengying Wang, Zongmu Xiao, Yixin Zhang

**Affiliations:** 1 Department of Energy and Material Engineering, Shandong Polytechnic College, Jining 272067, China; zhoujing_sdpu@163.com; 2 State Key Laboratory of Coking Coal Resources Green Exploitation, China University of Mining and Technology, Xuzhou 221116, China; qiqizhang@cumt.edu.cn (Q.Z.); wangmengying@cumt.edu.cn (M.W.); zongmuxiao@cumt.edu.cn (Z.X.)

**Keywords:** modified biochar, platinum–copper bimetallic catalyst, N-vinylformamide, hydrosilylation

## Abstract

The design and development of low-cost and efficient nonhomogeneous catalysts for silicon hydrogen addition is a hot research topic. This study reports the synthesis of a novel solid catalyst, PtCu-NVF-HBNC, derived from hazelnut shell biomass waste. The catalyst was prepared through carbonisation, nitrogen-doped activation, functionalisation with N-vinylformamide (NVF), and subsequent bimetallic Pt–Cu loading. It features a divalent platinum–vinylamine complex as the active centre. The introduction of Cu as a promoter induces competitive coordination with the Pt(II)–vinylamine complex, leading to electron density redistribution on the vinylamine ligand and a significant enhancement in Pt(II) coordination bond strength. This electronic modulation results in markedly improved activity and regioselectivity in the hydrosilylation of alkenes with tertiary silanes. The optimised catalyst, Pt_1.6_Cu-NVF-HBNC, demonstrated high performance in the hydrosilylation of linear alkenes, including 1-hexene, 1-octene, and 1-octadecene with triethoxysilane, indicating broad substrate adaptability.

## 1. Introduction

The silane hydrogenation reaction of olefins is one of the important industrial processes, and the resulting alkyl silane coupling agents are used to produce high value-added silicone-based materials and fine chemical synthesis intermediates and are widely used in the manufacture of silane coupling agents and organosilicones [1,2,3]. Hydrosilylation catalysts can be classified into two types: homogeneous and heterogeneous catalysts. Homogeneous catalysts, due to their full contact between active species and reactants, readily initiate catalytic reactions and exhibit relatively high catalytic efficiency. However, they also suffer from drawbacks such as difficulty in separation, recovery, and reuse, as well as high costs for product purification. As a result, research on heterogeneous catalysts has become increasingly extensive. At present, the catalysts applied in silane hydrogen addition are mainly composed of organic ligands and noble metals, such as Pt [4,5], Ru [6], Pd [7], Rh [8], etc., and in addition, non-precious metal catalysts have also been studied accordingly [9], such as Fe [10], Co [11], and Ni [12]. However, the hydrosilylation of olefins requires catalysts with high activity and regioselectivity, and side reactions such as hydrogenation, dehydro-silanation, and isomerisation of alkylsilanes and olefins often occur, so the commonly used catalysts in the hydrosilylation of olefins are still Pt catalysts [13]. However, these catalysts generally suffer from problems such as difficulty in separating from the reaction system after use, high cost and poor catalytic selectivity [14,15,16], so it is crucial to develop a non-homogeneous Pt-based catalyst with high activity and high regioselectivity.

Recent studies on bimetallic catalytic systems have achieved notable progress, primarily focusing on two structural types: one is homogeneous coordination compound catalysts, such as [Rh(η^3^-C_11_H_7_PO)(CO)(PPh_3_)], and the other involves bimetallic nanoparticles directly loaded onto supports, such as PdCu-SiO_2_ and nano-PtNi/NC-1000. Experimental data confirm that, compared to monometallic catalysts, bimetallic systems exhibit significant performance advantages. This enhancement primarily stems from two mechanisms: First, the synergistic interaction between bimetallic components can form structurally stable heteronuclear coordination compounds, whose unique electronic structures and geometric configurations optimise reaction pathways. Second, the interaction between metals effectively inhibits the aggregation of active components, significantly improving their dispersion on the support surface. These structural characteristics collectively enhance the catalytic performance of bimetallic systems, demonstrating superior reactivity and stability.

Among Pt-based bimetallic catalysts, PtCu nanocatalysts have attracted considerable attention due to the unique electronic effects between Pt and Cu, which effectively lower the d-band centre of Pt, thereby suppressing Pt oxidation. Additionally, Cu itself is a promising catalytic metal. Despite advances in the synthesis and application of bimetallic nanoparticles, developing reliable technical methods to produce catalysts with high-loading precious metal nanoparticles on carbon supports remains a challenging problem.

The preparation of a range of non-homogeneous catalysts applied to catalyse the silyl hydrogenation reaction of olefins by loading metals onto carriers has received much attention. Many substrates have been used as carrier materials, like metal oxides [17], silica molecular sieves [18], oxide graphite [19], metal–organic frameworks (MOFs) [20] and so on [21]. Compared with other materials, porous carbon, which is renewable and rich in pores, amorphous structure, and a large number of off-domain π-bonds, can be further modified by convenient methods and is considered to be a unique carrier for the recovery of precious metals through combustion [22,23]. And the food industry wastes as biomass carbon materials in various applications are increasing [24]. It was found that the catalytic activity, regioselectivity or stability of the Si hydrogenation reaction could be improved by adding additives, modifying the catalyst carrier and designing ligands for rational modification [25,26,27,28]. However, the existing non-homogeneous catalysts still suffer from low catalytic activity, regional selectivity and poor stability, and new modification methods need to continue to be explored. N-vinylformamide (NVF) is a selectively structurally stable aqueous polymer containing a large number of hydrogen bonding sites with a large number of cations that can influence the electronic structure of Pt nanoparticles [29,30].

This study developed a heterogeneous catalyst through nitrogen-doped modified hazelnut shell carbon for catalysing the hydrosilylation of three alkenes with triethoxysilane (TES). By introducing NVF ligands, its vinyl and amino functional groups effectively chelated and immobilised Pt-Cu bimetallic active components. The resulting PtCu-NVF-HBNC catalyst exhibited high catalytic activity, excellent regioselectivity, and remarkable stability under solvent-free and mild conditions, with well-dispersed metal particles and no aggregation. Simultaneously meeting low-cost and sustainability requirements, this catalyst demonstrates significant potential for industrial applications.

## 2. Materials and Methods

### 2.1. Materials and Apparatus

The raw materials used in the experiments were hazelnuts originating from Anshan City, China. N-vinylformamide (NVF, 96%); chloroplatinic acid hexahydrate (H_2_PtCl_6_·6H_2_O, Pt ≥ 37.5%); copper(II) nitrate trihydrate(Cu(NO_3_)·3H_2_O, ≥99%); N-methyl-2-pyrrolidone (NMP, >98%); urea (85%); zinc chloride (ZnCl_2_, ≥99.8%); 1-hexene (95%); 1-octence (purity 99.5%); 1-octadecene, purity 95%; triethoxysilane (TES, purity 97%); and other reagents were purchased from Aladdin (Shanghai, China).

### 2.2. Preparation of Catalysts

#### 2.2.1. Carriers Preparation (HBC, HBNC, and NVF-HBNC)

Hazelnut shells collected were prepared by calcination to produce biomass charcoal (HBC), which was then activated and treated with urea to obtain N-doped biochar (HBNC). HBNC was then modified with NVF to successfully graft a sufficient number of vinylamino ligand groups on the surface of HBNC to anchor the metal active ingredient (referred to as NVF-HBC). Detailed preparation conditions are described in the Supplementary Materials.

#### 2.2.2. Catalysts Preparation and Test

Preparation of Pt metal and Cu metal loaded NVF-HBNC (PtCu-NVF-HBNC). The obtained NVF-HBNC (0.5 g) was mixed with 30 mL of anhydrous ethanol and sonicated for 5 min. Keeping the sonication, 1.5 mL of 0.1 g/mL H_2_PtCl_6_·6H_2_O solution was added at a rate of 0.05 mL·s^−1^. Then 0.02 g NaHCO_3_ was added. After the mixture was mechanically stirred for 3 h at 60 °C, an appropriate amount of 0.1 g/mL Cu(NO_3_)·3H_2_O solution was also put in the above mixture at a rate of 0.05 mL·s^−1^. Mechanically stirred for 3 h at 80 °C to fully anchor the Pt and Cu in the precursor to the substrate. After the impregnation treatment, the black solid product was obtained by filtration and then washed with anhydrous ethanol and deionised water alternately and dried under vacuum for 12 h at 60 °C. The product was then dried under vacuum for 3 h at 60 °C.

In order to systematically investigate the effects of carrier properties and ligand modification on the catalyst structure, three control samples were prepared simultaneously: PtCu-HBC with unmodified carrier directly loaded with platinum, PtCu-HBNC with nitrogen-doped carrier loaded with platinum, and PtCu-NVF-HBC modified with ligand but not nitrogen-doped.

The loading amounts of different metal components on the as-prepared catalysts were all determined by the ICP-MS method elucidated in Supplementary Materials.

### 2.3. Catalytic Applications

**Figure 1:**
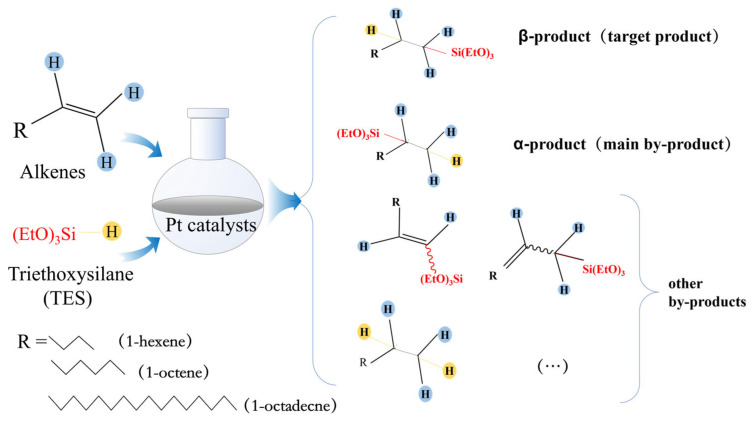
Selected hydrosilylation of representative alkenes with TES to evaluate catalytic performance.

The catalytic performance of five prepared non-homogeneous catalysts was compared under the same reaction conditions. In a three-necked flask with a reflux condenser tube and a dropping funnel, 5 mmol of 1-octene and 50 mg of biochar catalyst were added, put into an oil bath with stirring to raise the temperature, and then 5 mmol of TES was added slowly dropwise with stirring to heat up the resulting product to isolate the solid catalyst (Figure 1).

## 3. Results and Discussion

### 3.1. Physicochemical Properties and Structural Analysis of Different Carriers

**Figure 2:**
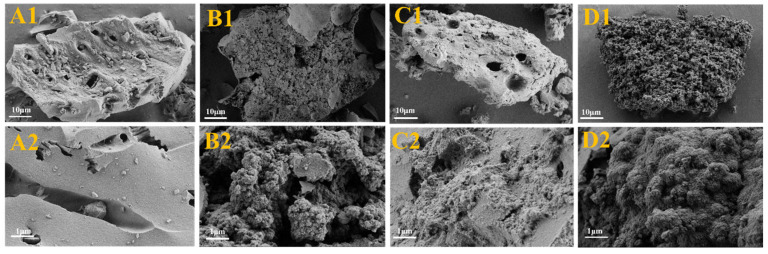
SEM of HBC (**A1**,**A2**), HBNC (**B1**,**B2**), NVF-HBC (**C1**,**C2**), and NVF-HBNC (**D1**,**D2**).

In this study, the surface morphology and structural features of HBC, NVF-HBC, HBNC and NVF-HBNC biochar carriers were analysed using scanning electron microscopy (SEM), and the results are shown in Figure 2. As can be seen from the figure, after nitrogen doping the surface of HBNC (Figure 2A) is rougher and forms spherical stacks of various sizes to obtain a larger specific surface area. In addition, the surface of NVF-HBC (Figure 2C) and NVF-HBNC (Figure 2D) obtained after modification by NVF was also attached with fibrous structures, confirmation of the successful loading of amine-based structures onto the surface of biomass porous carbon. The pore structure was tightly connected together, which reduced the specific surface area but made the porous carbon surface structure more compact, and this improvement was conducive to the improvement of the loading performance on the metal and further optimised the catalytic application of it. These filamentous structures provided favourable conditions for the loading of Pt.

**Figure 3:**
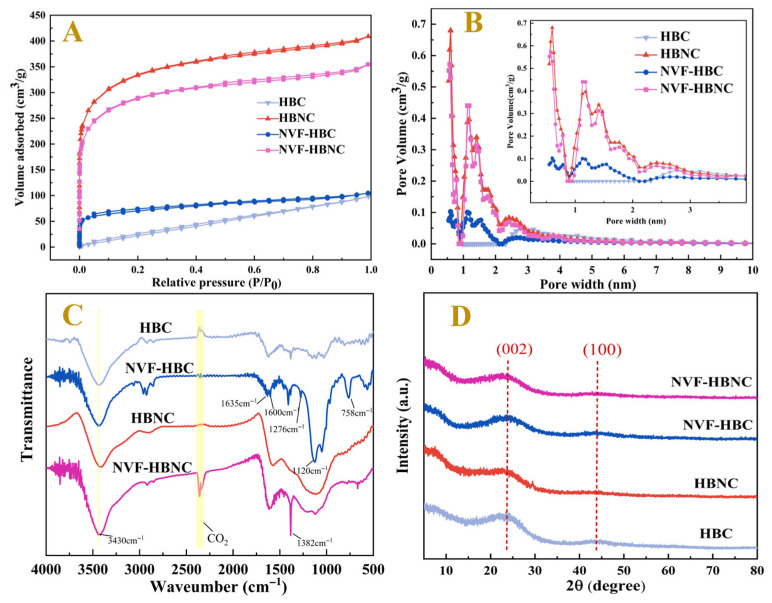
Spectral characterisation of the HBC, NVF-HBC, HBNC, and NVF-HBNC, N_2_ adsorption–desorption isotherm (**A**) and pore size distribution (**B**), FT-IR (**C**), and XRD (**D**).

In order to demonstrate again the effects of NVF modification and the introduction of nitrogen heteroatoms on the improvement of the pore structure of porous carbon, N_2_ adsorption-desorption tests were performed on HBC, NVF-HBC, HBNC and NVF-HBNC. The pore size distribution curves, pore sizes and BET surface areas calculated from the adsorption isotherms are shown in Figure 3 and Table 1. The test results for all four samples show typical type I (Figure 3A) adsorption isothermal curves, characterised by a wider range of pore size distributions in the material, including wider micropores and possibly narrower mesopores [31], indicating the existence of microporous and mesoporous structures within the porous carbon materials. The specific surface area of the HBNC sample was 1215.297 m^2^g^−1^ and the total pore volume was 0.548 cm^3^g^−1^, which was significantly better than that of HBC, indicating that N doping can promote the development of the charcoal pore structure, and these pores can facilitate the entry of metal ions into the interior of the samples, resulting in an increase in metal loading [32]. However, the specific surface area of NVF-HBNC after NVF modification decreased to 1054.483 m^2^g^−1^ and the total pore volume decreased to 0.49 cm^3^g^−1^, which reconfirmed that the loading of amine groups with fibrous structure onto the surface of biochar, inhibited the occurrence of side reactions in hydrosilylation and prevented the loss of metals in the catalytic process [33].

By comparing the FTIR spectra of different catalyst carriers, HBC, NVF-HBC, HBNC and NVF-HBNC, as shown in Figure 3C. The peaks observed at 3430 cm^−1^ corresponded to the O-H/N-H stretching vibration peaks as well as the C-H stretching vibration. Upon the introduction of NVF, the characteristic absorption peaks of the -OH bond broadened and shifted due to the incorporation of the stretching vibration absorption peaks of the -NH bond. In NVF-HBC and NVF-HBNC, 1630 cm^−1^ belongs to the stretching vibration of C=C bonds, and 1382 cm^−1^ corresponds to the C-N stretching vibration peaks of amine, the bending vibration of C-H bonds, and the -NO_2_ stretching vibration. These spectral analyses reconfirmed the successful introduction of vinyl amine groups into biochar after the addition of NVF. Meanwhile, the N atoms on the doping have empty orbitals, which can produce electronic effects with their coordinated Pt atoms, so that the catalyst exhibits high activity.

The crystal structures and the degree of disorder or defects of the four samples were investigated by the XRD technique. As shown in Figure 3D, two relatively broad diffraction peaks at 24° and 43° correspond to the (002) and (100) planes of the amorphous carbon layer, respectively, indicating that the four porous carbon materials belong to the amorphous structure [34]. HBC, HBNC and NVF-HBC have stronger (002) planar intensity ratios than that of NVF-HBNC, and the latter is also characterised by a broadening of the graphite peak, suggesting that the presence of urea-doped N atoms and the NVF modifier effectively inhibits the graphitisation of carbon and increases the disordered structure of the porous carbon material. This disordered structure enables NVF-HBNC to obtain a high specific surface area and pore volume, which is useful for improving the metal loading capacity of NVF-HBNC.

**Table 1:** Pore structure characteristics of the HBC, NVF-HBC, HBNC, and NVF-HBNC.

Sample	S_BET_ [m^2^g^−1^]	V_total_ [cm^3^g^−1^]	V_micro_ [cm^3^g^−1^]	V_meso_ [cm^3^g^−1^]	D_ave_ [35]
HBC	129.85	0.135	0	0.135	4.697
NVF-HBC	254.102	0.143	0.092	0.051	2.553
HBNC	1215.297	0.548	0.418	0.13	2.082
NVF-HBNC	1054.483	0.49	0.37	0.12	2.081

**Figure 4:**
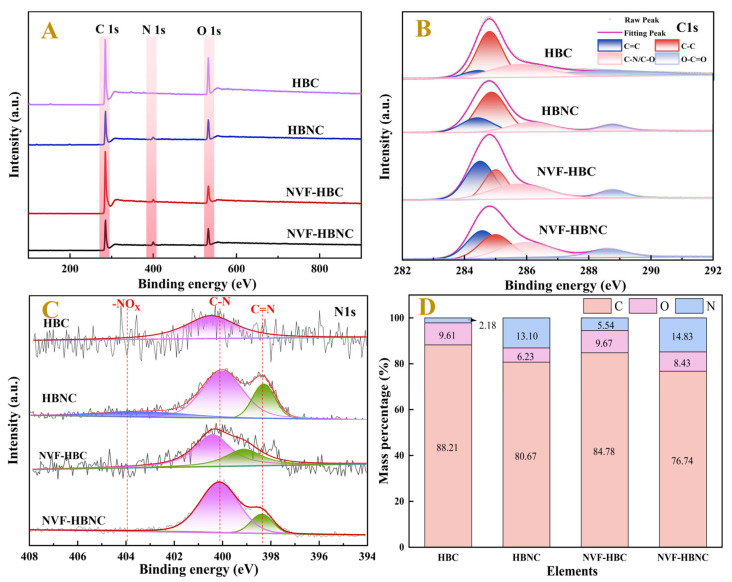
Spectral characterisation of supports, XPS spectra (**A**), C1s (**B**), N1s (**C**), and elemental composition (**D**).

Figure 4 shows the XPS spectra of HBC, HBNC, NVF-HBC, and NVF-HBNC, which show three distinct peaks at binding energies of 248.8 eV, 533.0 eV, and 400.2 eV belonging to the C1s, O1s, and N1s peaks, respectively. Figure 4B,C demonstrate the fitted plots of the fine scanning spectra of C1s and N1s for the four samples. Figure 4B demonstrates three independent sub-peaks of the fitted C1s spectra, 284.4 eV, 284.8 eV, and 286.3 eV. Out of the peaks are from C=C, C-C, and C-O/C-N bonds in the carbon materials, respectively [36]. The change in the area of these characteristic peaks reveals a slight increase in the content of C=C bonds after N doping and a significant increase in C=C bonds after modification with N-vinylformamide and a significant increase in the content of C-O/C-N bonds. Figure 4C High-resolution N1s spectra are classified into pyridine nitrogen C=N (398–399 eV), pyrrole nitrogen C-N (399–401 eV) and -NO_2_ (401–404.5 eV). The change of these characteristic peaks can indicate that the molecular structure was not damaged after N doping, and the vinylamine group was successfully grafted on the surface of hazelnut shell carbon after NVF modification, which can form Pt(II)-vinylamine coordination compounds [28].

The C, O and N contents of the four samples were determined by elemental analyser as shown in Figure 4D, which shows that HBC has been activated and modified to successfully introduce a large number of N-/O-containing groups. The comparison between HBNC and NVF-HBC shows that NVF has been effectively introduced to the surface of HBNC. The abundance of nitrogen and oxygen functional groups can be used as active sites to accelerate catalytic reactions [37].

A series of catalysts were obtained by varying the preparation parameters, including the amount of precursor and different carriers, according to the preparation method in Section 2.2. The mass ratio of the two metal components loaded on the catalysts was determined by the ICP-MS method. The results are summarised in Table 2. In addition, the obtained catalysts were numbered and named to facilitate subsequent analysis and discussion.

**Figure 5:**
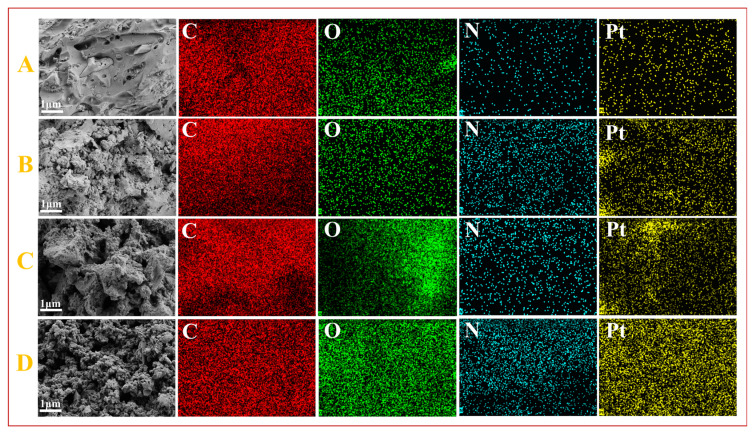
SEM images and corresponding EDS elemental mappings of Pt-HBC (**A**), Pt-NVF-HBC (**B**), Pt-HBNC (**C**), and Pt-NVF-HBNC (**D**).

Figure 5 compares the SEM and EDS elemental mapping of the four catalysts, which again demonstrates that the content of O and N elements increased significantly after N doping and NVF modification, and there was no significant change in the catalyst morphology after loading of metal Pt in comparison with Figure 2. It can be observed that the O and N elements have good dispersion on top of the carrier, while the Pt is uniformly distributed on the porous carbon carrier, and no agglomeration of the metal is observed.

**Figure 6:**
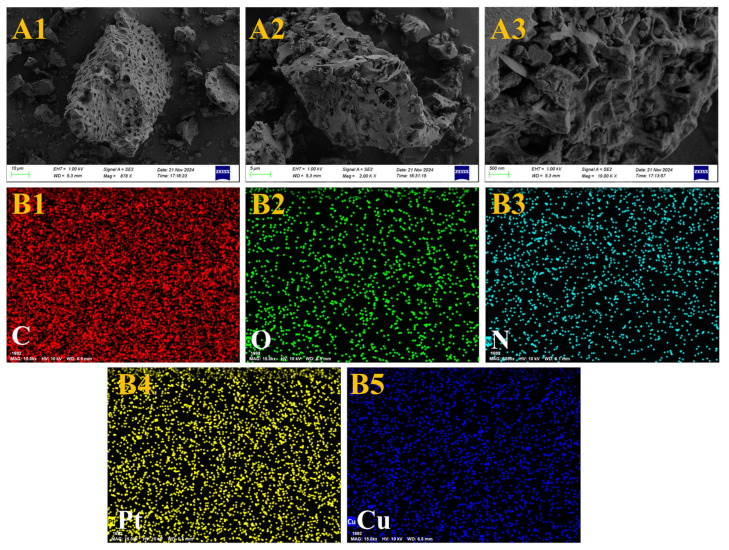
SEM (**A1**–**A3**) and EDS (**B1**–**B5**) of Pt_1.6_Cu-NVF-HBNC.

Compared with the prepared Pt-NVF-HBNC catalyst, the introduction of Cu as a promoter did not significantly alter the morphology of the biochar, which maintained its original porous structure, as shown in Figure 6. However, notable differences emerged in surface details, exhibiting a substantial number of metallic nanoparticles. This phenomenon arises from the successful reduction of both Pt and Cu noble metal elements to nanoscale dimensions during the preparation process, followed by their composite adsorption onto the biochar surface. The presence of these metallic nanoparticles enriches the microscopic morphology of the catalyst surface and provides additional active sites for reactions. Further EDS analysis confirmed the relatively uniform distribution of Pt and Cu metallic nanoparticles on the carbon surface. This characteristic enables enhanced utilisation of surface elements during catalytic reactions, thereby improving both reaction selectivity and catalytic activity. Moreover, the incorporation of Cu resulted in the dispersion of variably sized metal particles across the originally uniform carbon layers, while Pt elements were uniformly embedded within these metal particles, collectively forming the unique structure of the catalyst. Consequently, the Pt_1.6_Cu-NVF-HBNC catalyst prepared in this study not only preserves the biochar morphology but also benefits from the abundant metallic nanoparticles and their homogeneously distributed elemental properties, laying a solid foundation for enhanced catalytic performance.

**Figure 7:**
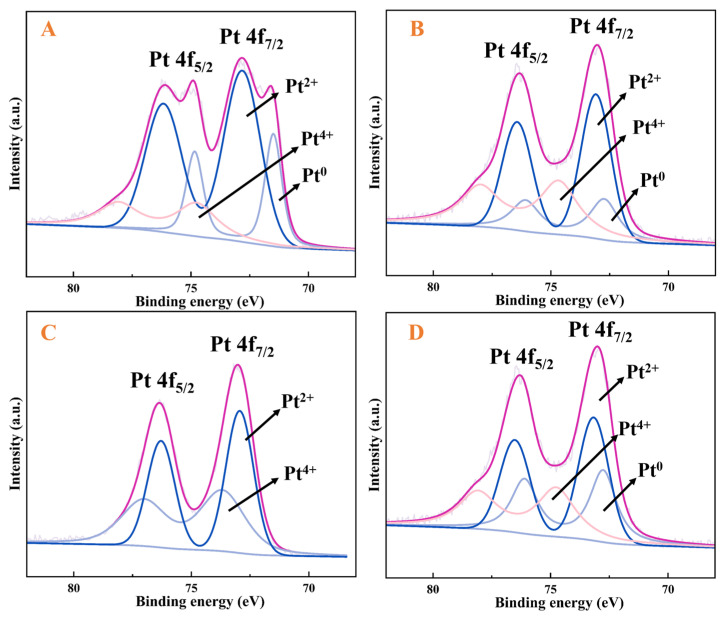
Pt4f spectra of Pt_0.23_Cu-NVF-HBNC (**A**), Pt_1.1_Cu-NVF-HBNC (**B**), Pt_1.6_Cu-NVF-HBNC (**C**), and Pt_3.2_Cu-NVF-HBNC (**D**).

XPS analysis was employed to determine the chemical composition and electronic structure of the catalyst surface. Deconvolution fitting of Pt4f spectra was performed for four catalysts with different Pt/Cu ratios, as shown in Figure 7. The XPS characterisation analysis of Figure 7 confirms that in the Pt_x_Cu-NVF-HBNC catalyst system, platinum predominantly exists in the divalent state (Pt(II)), with its characteristic binding energy peak at 72.4 eV corresponding to the Pt4f_7_/_2_ orbital [38]. Comparative XPS analysis of bimetallic catalysts with varying Pt/Cu mass ratios revealed that as the relative Pt content increases, the peak area of Pt4f_7_/_2_(II) exhibits an enhancement trend [39]. This phenomenon originates from the coordination interaction between vinyl functional groups modified on the carrier surface and Pt(IV) ions in the platinum precursor, which promotes the valence reduction of Pt(IV) and ultimately leads to the formation of a stable Pt(II)-vinylamine coordination structure. According to the described preparation protocol, the catalyst synthesis process can be divided into two critical steps: first, the construction of a Pt(II)-vinylamine coordination structure on the biochar carrier surface, followed by the introduction of Cu(II) ions that compete with the pre-formed Pt(II) complexes for vinyl coordination sites. This competitive process drives electron transfer toward Cu(II), resulting in the formation of complexes with unique dual-electron structures, which fundamentally causes the further increase in the Pt4f_7_/_2_(II) peak area. The study also demonstrated that the loading amount of the copper component requires precise regulation. Excessive copper content disrupts the original Pt(II) coordination structure, leading to an enlargement of the Pt4f_7_/_2_(IV) peak area and consequently significantly deteriorating the catalytic performance. Conversely, insufficient copper content results in weak competitive coordination capability, making it difficult to form an ideal bimetallic synergistic structure.

In summary, vinyl amino groups were stably immobilised on the HBNC surface by NVF modified. Then the Pt(IV) ions in the precursor can be effectively trapped and immobilised by the ligand groups introduced on the HBNC surface, resulting in the successful construction of a stable Pt(II)-vinylamine complex with a unique structure on the NVF-HBNC surface. Moreover, an appropriate amount of Cu(II) forms stable bimetallic active centre structures by sharing vinylamine-based ligands with Pt(II). This unique coordination mode not only optimises the electronic states of the metals but also significantly enhances the catalytic stability.

### 3.2. Catalytic Activity and Selectivity Studies

To evaluate the catalytic performance of the catalysts prepared in Table 2, reactions were conducted under solvent-free conditions at atmospheric pressure using 5 mmol of 1-octene, TES, and 50 mg of catalyst. The conversion of 1-octene and the content of β-addition products in the hydrosilylation reaction products were investigated by gas chromatography. The influence of the Pt/Cu ratio on the catalytic reaction was systematically analysed.

As shown in Table 3, the catalytic performance results of nine catalysts demonstrate that Pt_1.6_Cu-NVF-HBNC exhibits the most outstanding catalytic efficiency in the hydrosilylation reaction of 1-octene with triethoxysilane (TES), achieving conversion and selectivity rates of 99.2% and 99.4%, respectively, slightly higher than those of the monometallic Pt-NVF-HBNC. Notably, the monometallic Cu-NVF-HBC showed no catalytic activity in this reaction system, confirming that the copper–vinylamine complex structure lacks the capability to catalyse such reactions. Comparison among three catalysts with different Cu loadings reveals that the Pt/Cu mass ratio significantly influences catalytic performance. The bimetallic catalyst Pt_1.6_Cu-NVF-HBNC demonstrates optimal activity and selectivity, indicating an ideal mass ratio of 1.6:1.

This study systematically investigated the catalytic performance of the Pt_1.6_Cu-NVF-HBNC catalyst in olefin hydrosilylation reactions and its influencing factors. Experimentally, 1-hexene, 1-octene, and 1-octadecene were selected as representative substrates for short-chain and long-chain terminal olefins. Based on the boiling point characteristics of 1-hexene (62.8 °C), reactions were conducted at three temperature gradients: 40 °C, 50 °C, and 60 °C. For 1-octene and 1-octadecene, temperatures were set at 80 °C, 90 °C, and 100 °C, with time intervals ranging from 5 min to 3 h to investigate the reaction kinetics. 5 mL of olefin and TES were selected to study the effect of temperature and time on the reaction under 50 mg of Pt_1.6_Cu-NVF-HBNC at atmospheric pressure without solvent. The structures and contents of the chemical components in the products were determined by a combination of gas chromatography and ^1^H NMR. Figure S1 shows and analyses typical ^1^H NMR spectra of three hydrosilylation reaction products catalysed by Pt_1.6_Cu-NVF-HBNC at 90 (50) °C for 3 h. It should be noted that the activity and selectivity of the catalyst are measured by the olefin conversion and β-adduct content in the reaction products, respectively.

**Figure 8:**
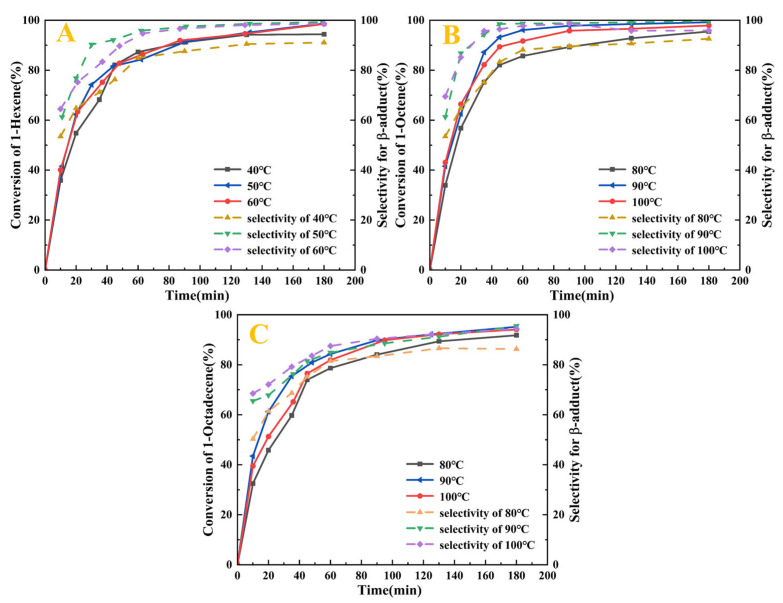
Catalytic activity of Pt_1.6_Cu-NVF-HBNC on three olefins: 1-hexene (**A**), 1-octene (**B**), and 1-octadecene (**C**).

As shown in Figure 8, Pt_1.6_Cu-NVF-HBNC demonstrates exceptional activity toward terminal alkenes, achieving >99.2% conversion and >99.6% β-selectivity for both 1-hexene and 1-octene. Notably, the reaction proceeds without an induction period, indicating rapid activation capability of the bimetallic Pt-Cu sites. For long-chain 1-octadecene, while maintaining 95.2% conversion, the selectivity decreases to 95.2% due to steric hindrance effects arising from the extended carbon chain. Temperature-dependent studies reveal a kinetic-to-thermodynamic control transition within the 80–90 °C range, where β-selectivity dramatically improves from <90% to >99%. Chronometric experiments confirm complete reaction within 3 h for all substrates, with longer-chain alkenes requiring extended duration for full conversion. These results demonstrate that substrate steric effects predominantly govern selectivity in this catalytic system.

**Figure 9:**
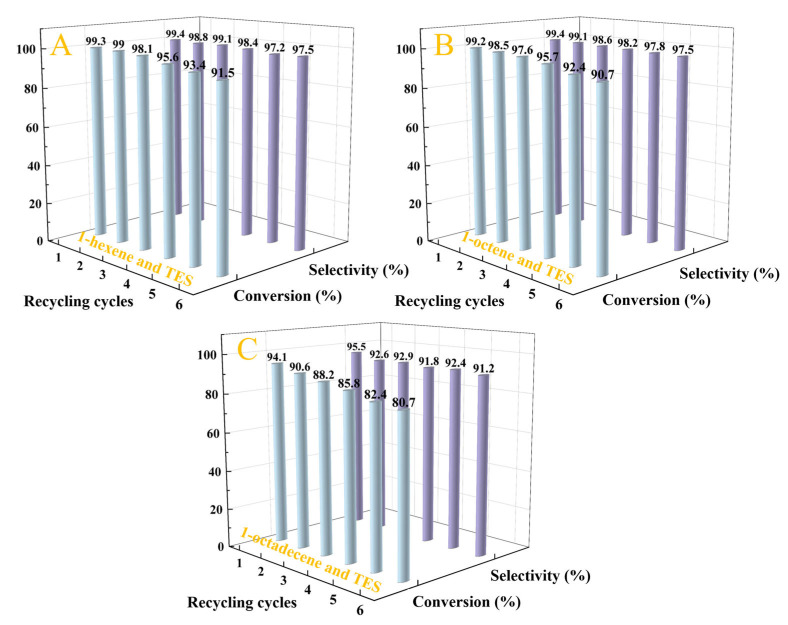
Stability of Pt_1.6_Cu-NVF-HBNC on three olefins: 1-hexene (**A**), 1-octene (**B**), and 1-octadecene (**C**).

The recycling performance of the Pt_1.6_Cu-NVF-HBNC catalyst in the hydrosilylation of TES with various alkenes was further investigated (Figure 9). Experimental results demonstrate exceptional stability of the catalytic material: after six consecutive reaction cycles, it maintains over 80% conversion efficiency for all three types of alkene substrates. This remarkable cycling stability primarily originates from the abundant vinylamine coordination groups on the carrier surface, which form stable coordination structures with metal components, effectively suppressing the leaching of active metal species. The Pt_1.6_Cu-NVF-HBNC catalyst not only exhibits excellent reusability but also retains high reactivity and regioselectivity throughout the cycling tests.

### 3.3. Kinetic Modelling

Kinetic research is of great theoretical value and practical significance in elucidating the reaction mechanism and guiding practical production [40]. This section systematically investigates the reactivity differences among various terminal alkenes by constructing kinetic models for the hydrosilylation reactions of three terminal alkenes with TES catalysed by Pt_1.6_Cu-NVF-HBNC, focusing on kinetic parameters such as reaction rate constants and activation energies. From the relevant information, the silicon hydrogen addition reaction involved in this experiment follows a kinetic secondary reaction process with the kinetic equation [41]:

(1) $$\frac{dx}{dt}=K_{A}{\left(a_{0}-x\right)}^{2}$$

where a_0_ is the initial concentration of the reactant, mmol·L^−1^; x is the concentration of the reactant at time t, mmol·L^−1^; t is the reaction time, min; K_A_ is the rate constant of the secondary reaction, (mmol·L^−1^·min)^−1^.

Integral deformation of Equation (1) yields the following equation:

(2) $$\frac{1}{a_{0}\left(1-r\right)}=K_{A}t+b$$

where (1 − r) is the rate of reactant conversion after reaction time t (r = x/a_0_); b is a constant.

The slope of the line obtained through linear regression analysis can be used to precisely calculate the second-order kinetic constant (KA) of different olefin substrates in the silyl hydrogenation reaction. The Arrhenius equation was then used to establish a quantitative correlation model between the temperature of the reaction system and the reaction rate constant. The specific derivation process is as follows:

(3) $$\ln K_{A}=\ln A-\frac{E_{a}}{RT}$$

where K_A_ is the reaction rate constant at different temperatures, (mmol·L^−1^·min)^−1^; A is the finger-forward factor (also known as the frequency factor); E_a_ is the apparent activation energy, J·mol^−1^; T is the thermodynamic temperature, K; and R is the molar gas constant, J·mol^−1^·K^−1^.

**Figure 10:**
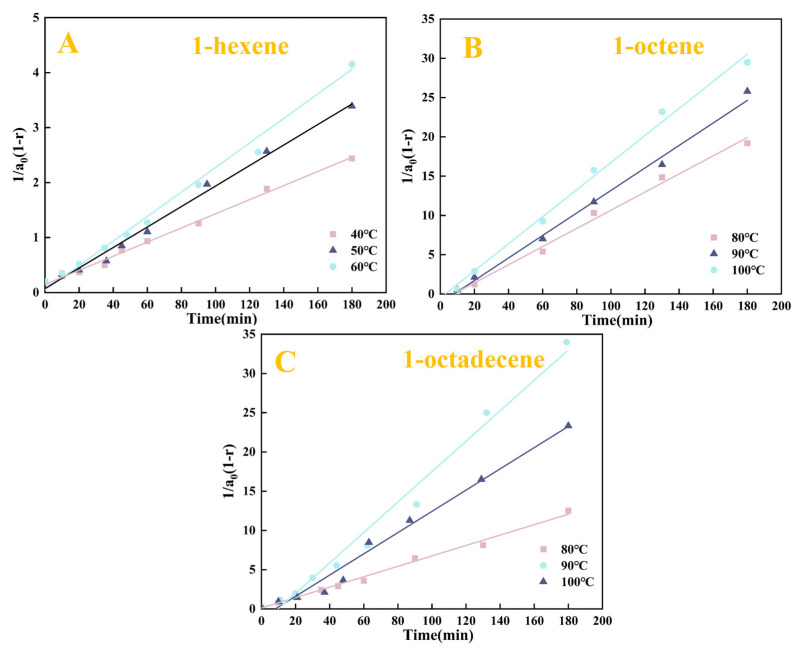
The plot of 1/[a_0_(1 − r)] versus reaction time (t) of Pt_1.6_Cu-NVF-HBNC: 1-hexene (**A**), 1-octene (**B**), and 1-octadecene (**C**).

Based on the kinetic Equation (2) and the experimental data of the Pt_1.6_Cu-NVF-HBNC catalysed reaction (Figure 8), this study constructed a functional relationship diagram of 1/[a_0_(1 − r)] as a function of time (Figure 10).

**Figure 11:**
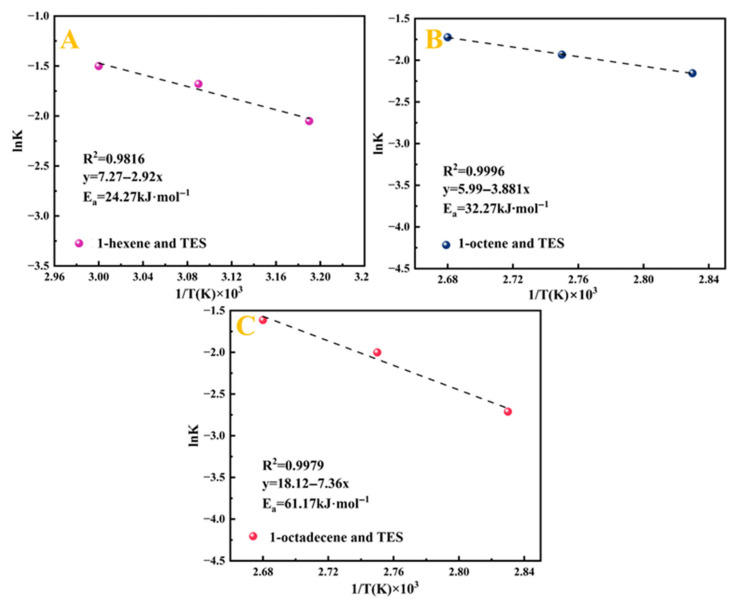
The Arrhenius plot of ln K versus 1/T × 10^3^ of Pt_1.6_Cu-NVF-HBNC: 1-hexene (**A**), 1-octene (**B**), and 1-octadecene (**C**).

Through linear regression analysis, the correlation coefficients (R^2^) and reaction rate constants (K_2_) under various temperature conditions were obtained. The relevant data are summarised in Table 4. To further investigate the effect of temperature on the reaction rate, based on the Arrhenius equation, the natural logarithm of the obtained rate constants (ln K) was correlated with the inverse of the absolute temperature (1/T × 10^3^). The resulting linear relationship is shown in Figure 11. A linear fitting plot was made with ln K_A_ versus T^−1^ (K^−1^), and the linear correlation coefficient R^2^ was greater than 0.9, which was a good fit.

The apparent activation energies required for the hydrosilylation of three terminal alkenes with TES catalysed by Pt_1.6_Cu-NVF-HBNC were determined from the slopes, and the second-order kinetic equations for the reactions of 1-hexene (Equation (4)), 1-octene (Equation (5)), and 1-octadecene (Equation (6)) with TES catalysed by Pt_1.6_Cu-NVF-HBNC were further derived according to Equation (3):

(4) $$\frac{dx}{dt}=1.43\times 10^{3}\exp\left(\frac{-24270}{RT}\right)\times {\left(a_{0}-x\right)}^{2}$$

(5) $$\frac{dx}{dt}=4\times 10^{2}\exp\left(\frac{-32270}{RT}\right)\times {\left(a_{0}-x\right)}^{2}$$

(6) $$\frac{dx}{dt}=7.27\times 10^{7}\exp\left(\frac{-61170}{RT}\right)\times {\left(a_{0}-x\right)}^{2}$$

The apparent activation energy (E_a_) is a critical factor determining reaction rates [42]. In this study, the constructed Pt(II)-vinylamine complex active centre forms stable interactions with TES, significantly reducing the reaction E_a_ and eliminating the induction period. Experimental data reveal an increasing trend in E_a_ with alkene chain length: 1-hexene (24.27 kJ·mol^−1^) < 1-octene (32.27 kJ·mol^−1^) < 1-octadecene (61.17 kJ·mol^−1^) [28]. The catalyst synergistically utilises steric and electronic effects to promote anti-Markovnikov addition of the Si-H bond to the alkene double bond, significantly enhancing selectivity toward β-addition products [43]. As the alkene chain lengthens, increased steric hindrance and enhanced double-bond stability raise the barrier for electrophile approach and reduce transition state stability, thereby requiring higher activation energy for long-chain alkenes [44]. The catalytic system follows the Chalk–Harrod mechanism, initially generating an electrophilic active intermediate, whose reactivity is markedly influenced by structural differences among alkenes.

### 3.4. Potential Catalytic Mechanisms

**Figure 12:**
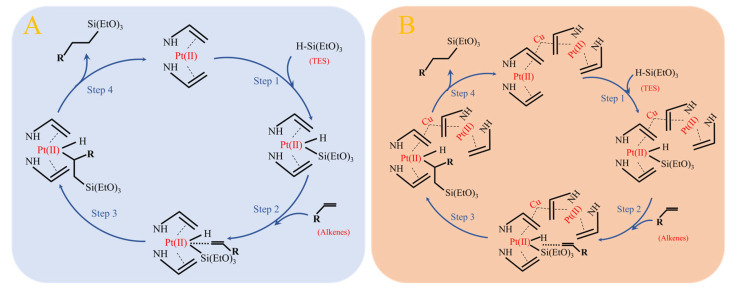
Possible catalytic mechanism of Pt-NVF-HBNC (**A**) and Pt_1.6_Cu-NVF-HBNC (**B**).

A proposed mechanism based on the literature precedents and our experimental observations. Based on the Chalk–Harrod reaction mechanism, the catalytic process can be divided into the following key steps [28]: First, the Pt(II) active centre on the catalyst surface undergoes oxidative addition with TES, forming an electrophilic active species with Pt(II) as the core (Step 1). Subsequently, this active species activates the Si-H bond and attacks the alkene double bond (Step 2), proceeding through addition (Step 3) and reductive elimination (Step 4) steps to ultimately generate the target product, as illustrated in Figure 12. Mechanistic studies reveal that the introduction of Cu(II) follows specific electronic modulation principles: it forms a stable bimetallic coordination structure with Pt(II), maintaining the divalent state of Pt through d-d orbital hybridisation without altering the electronic configuration of the active centre. This unique metal synergy aligns with Pauling’s electronegativity balance principle, endowing the catalyst with both high activity and stability. Simultaneously, the steric hindrance effect of the amine ligand adheres to fundamental molecular recognition rules, selectively suppressing side reaction pathways such as β-hydride elimination and enhancing regioselectivity to over 99%. The incorporation of an appropriate amount of Cu(II) establishes a Pt-Cu bimetallic coordination structure that preserves the valence state of Pt(II) and the formation of the final active species, maintaining Pt in its divalent state. Meanwhile, the introduced amine groups effectively inhibit side reaction active sites, thereby improving the regioselectivity of the target product. Preliminary mechanistic studies indicate that this conversion may involve a Pt/Cu bimetallic synergistic catalytic process rather than simple platinum nanoparticle catalysis. Research into the detailed mechanism of action of the catalyst is ongoing.

## 4. Conclusions

This study presents a methodology for the efficient valorisation of hazelnut shell waste by converting it into a nitrogen-doped activated carbon support, which was subsequently functionalised with N-vinylformamide and decorated with platinum–copper bimetallic sites. The resulting solid catalyst, PtCu-NVF-HBNC, exhibits remarkable performance in the hydrosilylation of alkenes with tertiary silanes under mild conditions. The synthetic approach enables high catalytic activity and excellent regioselectivity across a range of linear alkenes, including 1-hexene, 1-octene, and 1-octadecene, using triethoxysilane as the coupling partner. Preliminary mechanistic studies suggest that the transformation likely proceeds via a synergistic Pt/Cu bimetallic catalytic process rather than through simple platinum nanoparticle catalysis. Further investigations into the detailed reaction mechanism are currently underway. Future work will focus on scaling up the process to evaluate its practical applicability and expanding the substrate scope to include olefins bearing functional groups or steric constraints.

## Figures and Tables

**Table 2:** Nomenclature of different catalysts and the corresponding elemental composition.

Carrier	Volume of H_2_PtCl_6_·6H_2_O (mL)	Volume of Cu(NO_3_)_2_·3H_2_O (mL)	Mass Ratio (wt.%)		Mass Ratio of Pt:Cu	Name
			Pt	Cu		
NVF-HBNC	1.5	0.45	1.13	0.36	3.2	Pt_3.2_Cu-NVF-HBNC
NVF-HBNC	1.5	0.8	1.09	0.67	1.6	Pt_1.6_Cu-NVF-HBNC
NVF-HBNC	1.5	1.05	0.94	0.85	1.1	Pt_1.1_Cu-NVF-HBNC
NVF-HBNC	5	0	4.99	0	-	Pt-NVF-HBNC
NVF-HBNC	0	0.85	0	0.76	-	Cu-NVF-HBNC
HBC	1.5	0.8	0.89	0.58	1.53	Pt_1.5_Cu-HBC
HBNC	2.1	0.95	1.01	0.71	1.42	Pt_1.4_Cu-HBNC
NVF-HBC	1.5	0.8	1.03	0.62	1.66	Pt_1.7_Cu-NVF-HBC

**Table 3:** Comparison of catalytic performance of different catalysts for silicon hydrogen addition reaction.

Entry	Catalyst	Conv. (%)	Yield (%)
1	Pt_3.2_Cu-NVF-HBNC	95.1	95
2	Pt_1.6_Cu-NVF-HBNC	99.2	99.4
3	Pt_1.1_Cu-NVF-HBNC	91.2	93.6
4	Pt_0.23_Cu-NVF-HBNC	75.4	89.8
5	Pt_1.6_Cu-HBC	61.1	78.4
6	Pt_1.5_Cu-HBNC	69.5	81.8
7	Pt_1.7_Cu-NVF-HBC	84.6	90.2
8	Cu-NVF-HBNC	0	0
9	Pt-NVF-HBNC	98.6	99.3

**Table 4:** Correlation coefficient R^2^ and reaction rate constant K_2_(mol·L^−1^·min)^−1^.

Reactant	Temperature (°C)	Correlation Coefficient R^2^	Reaction Rate Constant K_2_ (mol·L^−1^·min)^−1^
1-hexene and TES	40	0.9853	0.1285
	50	0.9935	0.1864
	60	0.9845	0.2231
1-octene and TES	80	0.9926	0.1157
	90	0.994	0.1447
	100	0.9939	0.1738
1-octadecene and TES	80	0.9911	0.0664
	90	0.9882	0.1353
	100	0.9852	0.1993

## Data Availability

The original contributions presented in this study are included in the article/Supplementary Materials. Further inquiries can be directed to the corresponding author.

## Supplementary Materials

The following supporting information can be downloaded at: https://www.mdpi.com/article/10.3390/molecules30183704/s1, Figure S1: The 1H NMR spectra of products 1-hexyltriethoxysilane (A), 1-octyltriethoxysilane (B) and 1-octadecyltriethoxysilane (C); Table S1: More details about the state of the art in this topic; Table S2: Pt_1.6_Cu-NVF-HBNC Platinum leaching during catalysis; References [45,46,47,48,49,50,51,52,53] are cited in the Supplementary Materials.
