# Supplementary material for: Construction of Pt-Cu-Vinylamine Complex on Hazelnut Shell Biochar as a Catalyst Used for Hydrosilylation of Alkenes by Tertiary Silanes
Source: Molecules. 2025 Sep 11;30(18):3704. doi: 10.3390/molecules30183704 (PMC12472727; doi:10.3390/molecules30183704)
Supplement: Supplementary file 1 [file molecules-30-03704-s001.zip › molecules-3800529-supplementary.pdf]

Supplementary Materials

For

Construction of Pt-Cu-Vinylamine Complex on Hazelnut Shell Biochar as a Catalyst Used for Hydrosilylation of Alkenes by Tertiary Silanes

Jing Zhou [1], Qiqi Zhang [2], Mengying Wang [2], Zongmu Xiao [2] and Yixin Zhang [2,*]

[1] Department of Energy and Material Engineering, Shandong Polytechnic College, Jining 272067, China; zhoujing_sdpu@163.com

[2] State Key Laboratory of Coking Coal Resources Green Exploitation, China University of Mining and Technology, Xuzhou 221116, China; qiqizhang@cumt.edu.cn (Q.Z.); wangmengying@cumt.edu.cn (M.W.); zongmuxiao@cumt.edu.cn (Z.X.)

* Correspondence: yixinzhang@cumt.edu.cn

Table of Contents

S1. Introduction (Table S1) 1

S2. Experimental Sections 2

S2.1 Preparation of carriers 2

S2.2 Synthesis procedures of comparison catalysts 2

S2.3 Catalyst activity test 3

S2.4 Characterization of catalyst 3

S2.5 Test of loading amount of Pt and Cu on catalyst 4

S3. Results and discussion 5

S3.1 Reaction product analysis (Figure S1) 5

S3.2 Catalyst reusability (Table S2) 6

References: 7

S1. Introduction

Table S1. More details about the state of the art in this topic

Catalysts	Olefins (mmol)	Silanes (mmol)[a]	Reaction temperature T (℃)	Reaction time t (h)	Solvent	Atmosphere	Conversion (%)
SiliaCat Pt(0)[1] (0.5 mol% Pt)	1-octene (5)	TES (6.5)	65	3	Toluene	Argon	93
SiliaCat Pt(0)[2] (0.025 mol% Pt)	1-hexene (150)	TES (100)	75	1	Solvent free	Air	100
	1-octene (150)						100
SiliaCat Pt(0)[2] (0.05 mol% Pt)	Styrene (100)	TES (125)	85	4			80
Pt-ISA/NG[3] (0.25 mol%Pt)	1-octene (1)	TES (1.3)	60	2	Solvent free	Air	95
Pt/SDB[4] (1.474×10^{-4} mmol%Pt)	10-bromo-1-decene (1.1)	$(HSiMe_2O)(i\text{-}Bu)_7Si_8O_{12}$ (1.1)	90	24	Toluene	Air	99
Pt LCSCs[5] (30 mg)	1-octene (6)	Dimethoxy-methylsilane (5)	60	20 min	Toluene	Air	92
SiO_2-DTPA-Pt[6] (2.8×10^{-3} mmolPt)	1-hexene (10)	Methyldichl-orosilane (18)	60	4	Solvent free	Air	99.6
$Pt_1^{\delta+}/TiO_2$[7] (1×10^{-3} mmolPt)	1-octene (1)	TES (1.1)	90	4	Toluene	Air	100
Pt-VTES-RSOC[8] (1×10^{-3} mmolPt)	1-hexene (5)	TES (5)	50	3	Solvent free	Air	99.3
	1-octene (5)						98.9
	Styrene (5)						97.8
PtNi-VTES-RSOC[9] (1×10^{-3} mmolPt)	1-octene (5)	TES (5)	50	3	Solvent free	Air	97.1

[a] TES: triethoxysilan

S2. Experimental Sections

S2.1 Preparation of carriers

Preparation of hazelnut shell biochar (HBC). Hazelnut shells were washed three times with deionised water to remove surface dust and impurities. The cleaned hazelnut shells were dried in an oven at 80 °C for 12 h and completely dried for subsequent use. Then, the hazelnut shell blocks were placed in a muffle furnace at room temperature and pyrolysed in an oxygen-deficient atmosphere with a temperature increase rate of 10 °C/min and a final temperature of 550 °C at a constant temperature for 1 h. After pyrolysis, the hazelnut shell biochars were ground using a ball mill and sieved through a 120 mesh (125 μm) sieve to obtain the biochar powder.

Preparation of nitrogen-doped hazelnut shell activated carbon (HBNC). Hazelnut shell powder and urea in a mass ratio of 1:1 were added to deionised water premixed with $ZnCl_2$ and stirred continuously for 12 h. The mixture was then poured into larger petri dishes and dried for 3 h at 80°C to remove water. It was then placed in a tube furnace and calcined at 600°C for 2 h with a heating rate of 5°C·min^{-1} and nitrogen atmosphere. Subsequently, the obtained powder samples were immersed in 2 M HCl solution with vigorous stirring. Finally, it was washed several times with deionised water to neutral and dried at 80°C for 12 h.

Preparation of NVF modified HBNC (NVF-HBNC). 2 g of HBNC and 50

mL of NMP were added to a round-bottomed flask and put into a magnetic stirrer for stirring, then 6 mL of NVF was added at a rate of 0.05 $mL{\cdot}s^{-1}$, and stirring was continued to mix homogeneously, and the temperature was slowly increased to 90°C with magnetic stirring for 7 h. The resulting product was washed in anhydrous ethanol and deionised water alternately, and dried under vacuum for 12 h at 60°C, which resulted in the modified NVF with NVF-HBNC sample. The vinyl groups contained therein can anchor metal ions by forming metal complex in the next synthetic applications, and the amino groups can improve the selectivity of the silylhydrogen addition reaction.

S2.2 Catalyst activity test

The catalytic performance of the non-homogeneous catalysts prepared as shown in Table 2 was investigated by the hydrosilylation of different olefins with triethoxysilane (TES). All the experiments were carried out in air under solvent-free conditions. The procedure was as follows: 5 mmol 1-hexene and 50 mg of catalyst were firstly added into a 10 ml flat-bottomed tube. The mixture was stirred (300 rpm) for 5 min and then 5 mmol of TES was added. Then, the mixture was heated at a set temperature and stirred (300 rpm) for an appropriate time. The resulting product was cooled in an ice water bath. The solid catalyst was separated by centrifugation. The components in the liquid product were determined by 1H nuclear magnetic resonance (1H-NMR) and the conversion and selectivity of olefins were determined by gas chromatography. The loading of Pt and Cu on the catalyst was measured by ICP-MS.

S2.3 Catalyst stability test

The stability of the prepared catalyst ($Pt_{1.6}$Cu-NVF-HBNC) was assessed by its reusability in the hydrosilylation reactions of TES with different alkenes. In

the first cycle, the adding amount of alkenes, TES and catalyst was consistent with that in Section S2.3 of SI. Selection of optimum conditions for reaction time and temperature. After the reaction, the liquid reactant was withdrawn by the centrifugation to analyze the component concentration by GC, while the catalyst used in the first cycle was left in the vessel bottom for the next cycle under the same reaction conditions. By analogy, the reusability of the catalyst in the hydrosilylation of TES with alkenes could be explored.

S2.4 Characterization of catalyst

The microstructure morphology of samples was observed by field emission scanning electron microscope (SEM, MAIA3 LMH, Tescan), its accompanying energy-dispersive X-ray spectrometer (EDS) was used to analyze elemental mapping. The Brunauer-Emmett-Teller method determined porosity properties with N_2 adsorption-desorption isotherms (BET, BELSORPmax ver 2.1, Ankersmid). The microstructure of the of carbon-rich zones was evaluated by Raman spectroscopy (Senterra R200-L, Bruker) with Ar-ion laser (532 nm) as the source. Fourier transform infrared spectrometer (FTIR, Tensor 27, Bruker) was applied to analyze the surface functional groups and chemical bonds of materials. Chemical state and composition were analyzed by X-ray photoelectron spectroscopy (XPS, ESCALAB 250Xi, Thermo) with an Al (Kα) source. X-ray diffraction (XRD) analysis was performed using a Bruker D8 ADVANCE diffractometer with a Cu (Kα) source. The chemical structure and concentration of the products were characterised using Gas chromatography-mass spectrometry (GC-MS,7890B-5977A, Agilent). The resulting products were analysed by NMR spectroscopy (Bruker Avancelll 600

MHz) to 1H NMR spectra, using deuterated chloroform as solvent. The data were analysed and compared by ChemDraw and MestReNove.

S2.5 Test of loading amount of Pt and Cu on catalyst

In the catalyst preparation process, 0.5 g of NVF-HBNC carrier was dispersed in 30 mL of anhydrous ethanol and sonicated for 5 min to form a homogeneous suspension. Under continuous sonication, 0.01 g/mL of chloroplatinic acid solution was first added dropwise at a constant rate of 0.05 mL/s. After sonication for 2 h, a predetermined amount of 0.01 g/mL of copper nitrate solution was added at the same rate. Sonication was continued for 5 minutes to ensure homogeneous mixing. The above mixed system was transferred to a 60°C constant temperature oil bath reaction unit and reacted under magnetic stirring conditions for 6 hours. Upon completion of the reaction, the solid product was separated by vacuum filtration and washed three times with anhydrous ethanol to remove unreacted metal salts. The residual reaction solution and the absolute ethanol wash were collected in a beaker and then the ethanol collected in the beaker was completely evaporated in a fume hood. The residue in the beaker was dissolved with 2 mL of 10M HNO_3 solution, and then an appropriate amount of water was added to bring the volume of the solution to 40 mL. The concentrations of Pt and Cu in the resulting 40 mL solution can be detected by ICP-MS to determine the amount of Pt and Cu in the residual reaction solution and the wash solution.

S3. Results and discussion

S3.1 Reaction product analysis

The ^{1}H NMR spectra were obtained on a Bruker AVANCEIII 600 MHz spectrometer, using $CDCl_3$ as solvent and tetramethylsilane as internal standard.

The ^{1}H NMR spectrum for the hydrosilylation product of 1-hence with TES at 50 ℃ and 3 h was shown in Fig. S1A: δ 0.57-0.64 (t, 2H, $(CH_3CH_2O)_3Si\text{-}\underline{CH_2}\text{-}$), δ 0.86 (t, 3H, $(CH_3CH_2O)_3Si\text{-}CH_2\text{-}(CH_2)_4\text{-}\underline{CH_3}$), δ 1.21 (t, 9H, $(\underline{CH_3}CH_2O)_3Si\text{-}CH_2\text{-}$), δ 1.25-1.42 (m, 8H, $(CH_3CH_2O)_3Si\text{-}CH_2\text{-}(\underline{CH_2})_4\text{-}$) and δ 3.80 (q, 6H, $(CH_3\underline{CH_2}O)_3Si\text{-}CH_2\text{-}$).

The ^{1}H NMR spectrum for the hydrosilylation product of 1-octene with TES at 90 ℃ and 3 h was shown in Fig. S1B:, δ 0.59-0.63 (t, 2H, $(CH_3CH_2O)_3Si\text{-}\underline{CH_2}\text{-}$), δ 0.86 (t, 3H, $(CH_3CH_2O)_3Si\text{-}CH_2\text{-}(CH_2)_6\text{-}\underline{CH_3}$), δ 1.21 (t, 9H, $(\underline{CH_3}CH_2O)_3Si\text{-}CH_2\text{-}$), δ 1.25 (d, 12H, $(CH_3CH_2O)_3Si\text{-}CH_2\text{-}(\underline{CH_2})_6\text{-}$) and δ 3.80 (q, 6H, $(CH_3\underline{CH_2}O)_3Si\text{-}CH_2\text{-}$).

The ^{1}H NMR spectrum for the hydrosilylation product of 1-octadecene with TES at 50 ℃ and 3 h was shown in Fig. S1C:, δ 0.45-0.70 (m, 2H, $(CH_3CH_2O)_3Si\text{-}\underline{CH_2}\text{-}$), δ 0.69-0.96 (m, 3H, $(CH_3CH_2O)_3Si\text{-}CH_2\text{-}(CH_2)_{16}\text{-}\underline{CH_3}$), δ 1.14 (d, 9H, $(\underline{CH_3}CH_2O)_3Si\text{-}CH_2\text{-}$), δ 1.25 (m, 32H, $(CH_3CH_2O)_3Si\text{-}CH_2\text{-}(\underline{CH_2})_{16}\text{-}$), and δ 3.56-3.92 (m, 6H, $(CH_3\underline{CH_2}O)_3Si\text{-}CH_2\text{-}$).

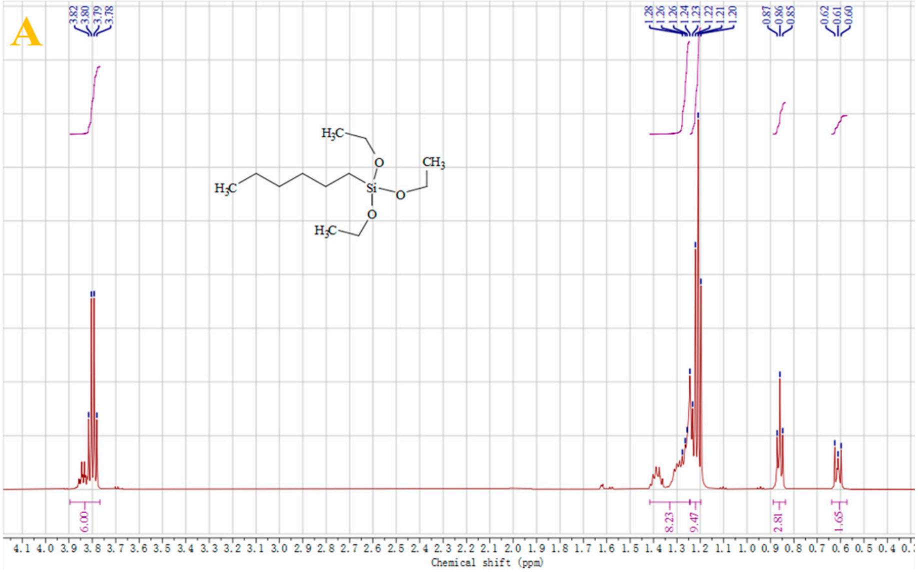
A
Chemical shift (ppm)

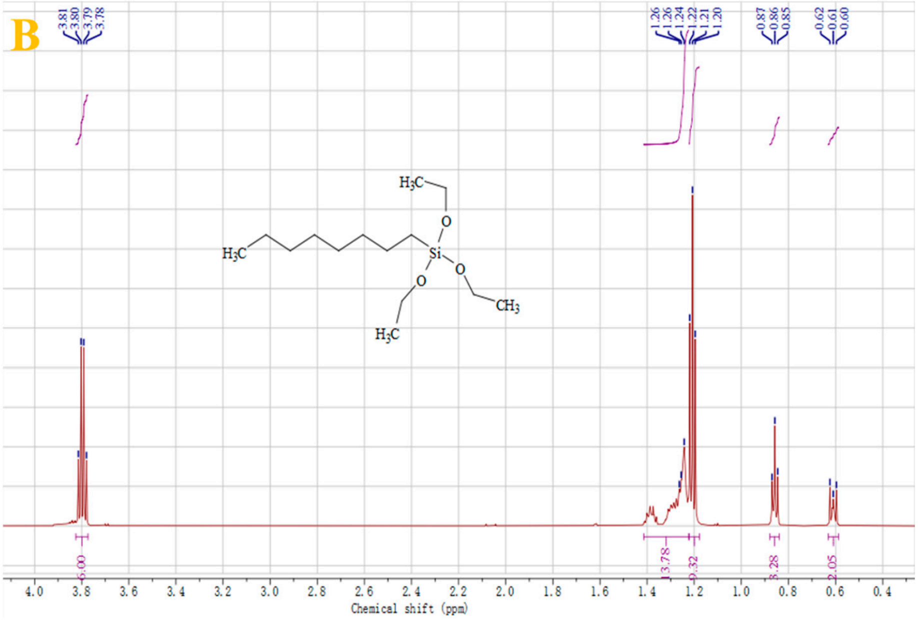
B
Chemical shift (ppm)

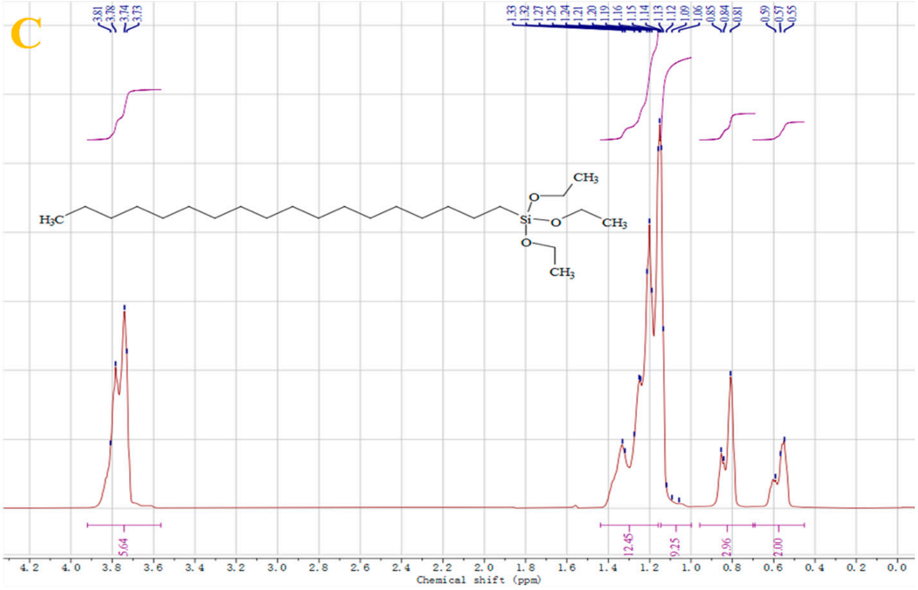
C
Chemical shift (ppm)

Figure S1. The 1H NMR spectra of products 1-hexyltriethoxysilane(A), 1-octyltriethoxysilane(B) and 1-octadecyltriethoxysilane(C)

S3.2 Catalyst reusability

The hot filtration method was employed to investigate the stability of the $Pt_{1.6}$Cu-NVF-HBNC catalyst in the hydrosilylation reaction of 1-octene with triethoxysilane (TES). The experiment was conducted at 90 °C, and the leaching concentration of Pt elements in the reaction system was monitored at different time points (0.5 h, 1 h, 2 h, 3 h) to evaluate the loss of the active Pt component.

The specific operational procedure was as follows: Under fixed reactant ratios (5 mmol 1-octene, 5 mmol TES) and catalyst dosage (50 mg), four parallel experimental groups were set up. Reactions were terminated at different time nodes, and the catalyst was separated. The Pt content in the reaction solution was then quantitatively analyzed using ICP-MS technique[10]. The volume of the reaction solution collected after centrifugal separation was kept constant at 5.0 mL. The leaching rate of the metal component was calculated according to the following formula:

$$\mathrm{R} = \frac{\mathrm{c} \times 5\mathrm{mL}}{c_0} \times 100\% \qquad (S1)$$

In the formula, R represents the leaching rate of platinum (%), c denotes the leaching concentration of platinum (μg/L), and c_0 indicates the initially added amount of platinum (mg).

Table S2. $Pt_{1.6}$Cu-NVF-HBNC Platinum leaching during catalysis

Time（h）	Leaching amount（μg/L）	Leaching rate（%）
0.5	12	0.008
1	43	0.03
2	74	0.052
3	98	0.069

As can be seen from the data in Table S2, in the hydrosilylation of 1-octene with triethoxysilane (TES) using $Pt_{1.6}$Cu-NVF-HBNC as the catalyst, the leaching rate of platinum after 3 hours of reaction did not exceed 0.1%. This indicates extremely low loss of the active Pt species, further demonstrating that

the structure formed by the coordination between platinum, copper, and the vinylamine group exhibits high stability and minimal leaching during the catalytic process. Consequently, the catalyst can maintain its activity effectively upon reuse.

References:

(1) Ciriminna, R.; Pandarus, V.; Gingras, G.; Béland, F.; Pagliaro, M. Closing the Organosilicon Synthetic Cycle: Efficient Heterogeneous Hydrosilylation of Alkenes over SiliaCat Pt(0). *ACS Sustainable Chemistry & Engineering* **2013**. DOI: 10.1021/sc3001096.

(2) Pandarus, V.; Ciriminna, R.; Gingras, G.; Béland, F.; Kaliaguine, S.; Pagliaro, M. Waste-free and efficient hydrosilylation of olefins. *Green Chemistry* **2019**. DOI: 10.1039/c8gc02569j.

(3) Zhu, Y.; Cao, T.; Cao, C.; Luo, J.; Chen, W.; Zheng, L.; Dong, J.; Zhang, J.; Han, Y.; Li, Z.; et al. One-Pot Pyrolysis to N-Doped Graphene with High-Density Pt Single Atomic Sites as Heterogeneous Catalyst for Alkene Hydrosilylation. *ACS Catalysis* **2018**. DOI: 10.1021/acscatal.8b02624.

(4) Walczak, M.; Stefanowska, K.; Franczyk, A.; Walkowiak, J.; Wawrzyńczak, A.; Marciniec, B. Hydrosilylation of alkenes and alkynes with silsesquioxane (HSiMe2O)(i-Bu)7Si8O12 catalyzed by Pt supported on a styrene-divinylbenzene copolymer. *Journal of Catalysis* **2018**. DOI: 10.1016/j.jcat.2018.08.012.

(5) Ali, I. S.; Chen, L.; Rezvani, F.; Zhou, X.; Tait, S. L. Tuning coordinated supported catalysts: Carboxylic acid-based ligands to improve ceria-supported Pt catalysts for hydrosilylation. *Applied Catalysis A: General* **2022**. DOI: 10.1016/j.apcata.2022.118634.

(6) Shao, D.; Li, Y. Preparation of polycarboxylic acid-functionalized silica supported Pt catalysts and their applications in alkene hydrosilylation†. *RSC Advances* **2018**. DOI: 10.1039/c8ra01828f.

(7) Chen, Y.; Ji, S.; Sun, W.; Chen, W.; Dong, J.; Wen, J.; Zhang, J.; Li, Z.; Zheng, L.; Chen, C.; et al. Discovering Partially Charged Single-Atom Pt for Enhanced Anti-Markovnikov Alkene Hydrosilylation. *Journal of the American Chemical Society* **2018**. DOI: 10.1021/jacs.8b03121.

(8) Wang, R.; Liang, S.; Zhu, J.; Luo, M.; Peng, H.; Shi, R.; Yin, W. Hydrosilylation of alkenes with tertiary silanes under mild conditions by Pt(II)-vinyl complex supported on modified rice straw biochar. *Molecular Catalysis* **2023**. DOI: 10.1016/j.mcat.2023.113141.

(9) Zhu, J.; Wang, R.; Zhang, S.; Xie, X.; Luo, M.; Peng, H.; Liu, Y.; Shi, R.; Yin, W. Supported bimetallic PtNi-vinyl complex for hydrosilylation of straight-chain terminal alkenes with tertiary silanes: Effect of Ni promoter on catalytic performance. *Chemical Engineering Science* **2024**. DOI: 10.1016/j.ces.2024.120228.

(10) Gärtner, D.; Sandl, S.; Jacobi von Wangelin, A. Homogeneous vs. heterogeneous: mechanistic insights into iron group metal-catalyzed reductions from poisoning experiments. *Catalysis Science & Technology* **2020**, *10* (11), 3502-3514. DOI: 10.1039/d0cy00644k.
